# Supplementary material for: Variability of functional and biodiversity responses to perturbations is predictable and informative
Source: Nat Commun. 2024 Nov 28;15:10352. doi: 10.1038/s41467-024-54540-3 (PMC11604961; doi:10.1038/s41467-024-54540-3)
Supplement: Supplementary file 1 — Supplementary Information [file 41467_2024_54540_MOESM1_ESM.pdf]

Supplementary Material for

Variability of functional and biodiversity responses to perturbations is predictable and informative

James A. Orr[1,2], Jeremy J. Piggott[3], Andrew L. Jackson[3],
Michelle C. Jackson[1] and Jean-François Arnoldi[4]

1. Department of Biology, University of Oxford, Oxford, UK.
2. School of the Environment, University of Queensland, Brisbane, Australia.
3. Zoology, School of Natural Sciences, Trinity College Dublin, Dublin, Ireland.
4. Centre National de la Recherche Scientifique, Experimental and Theoretical Ecology Station, Moulis, France.

Contents

1 Supplementary Note 1: Mismatches between diversity metrics 2

2 Supplementary Note 2: Validation of geometric approach 3

3 Supplementary Note 3: Collinearity of functions predicted by their estimated broadness 5

1 Supplementary Note 1: Mismatches between diversity metrics

Diversity metrics are non-linear functions, but from the angle between their gradients, we can still predict response mismatches. Here we compare metrics from the family of Hill diversity, which vary based on q, the hill exponent, which controls their sensitivity to common or rare species. Consequently, the difference in the q values between two diversity metrics correlates to the size of the angle between their gradients (Fig. S1A). The consistency of population-level responses has a weaker effect here due to the fact that diversity metrics are associated to non-positive functions. Therefore, a bias towards positive or negative population-level responses does not necessarily translate as a bias at the level of diversity measures. In fact, the evenness of the biomass distribution of a community determines how similar gradients of diversity metrics are to positive functions. For uneven communities, increasing the biomass of most species will increase diversity so gradients will behave similarly to positive linear functions. However, for perfectly even communities, increasing the biomass of any single species will decrease diversity so gradients of diversity will effectively be negative functions of biomass.

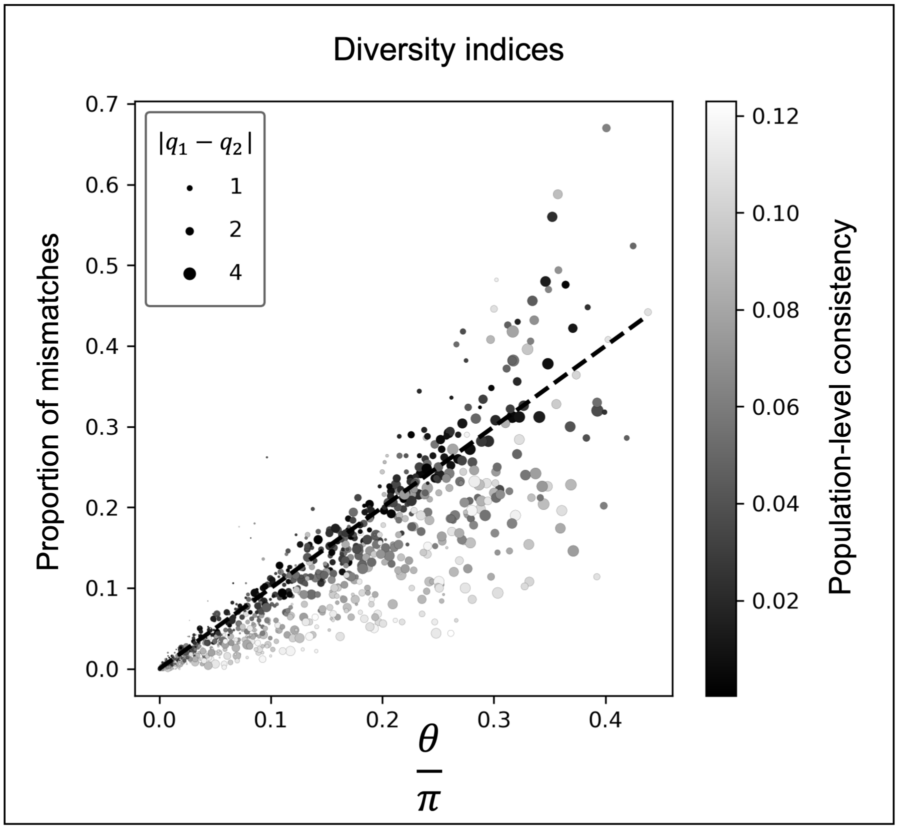

Figure S1: The proportion of mismatches between two diversity metrics is predicted by the angle between their gradient vectors. All diversity metrics came from the family of Hill diversity indices so increasing the difference between the q values of diversity indices (size of the points) increases the angle between their gradients, which therefore increases the proportion of mismatches.

2 Supplementary Note 2: Validation of geometric approach

In our formalization of response mismatches, an ecological function (such as biomass production, respiration, or enzyme concentrations related to nutrient cycling) is seen as a direction in the community state-space, so a unit vector

$$\boldsymbol{u} = (u_1, ..., u_S)$$

where the element u_i represents the (relative) effective per-capita functional contribution of species i (by effective we here mean that it could be scaled by its biomass elevated to the power α, when perturbations predominantly probe the contributions of the more abundant species). The number of species S could be unknown but assumed to be very large. We consider $K \ll S$ functions, so associated to K unit vectors $\boldsymbol{u}^{(1)}, ..., \boldsymbol{u}^{(K)}$, and compare the qualitative responses to perturbations of these functions to one-another. Ideally, when there is a high response diversity, and/or if species contribute in all kinds of way to the functions (so that their associated directions could point in any direction) we could use mismatch data of the form

$$0 \leq P_{ij} \leq 1,\ P_{ii} = 0,\ i, j = 1, ..., K$$

to get, after multiplication by π, the angle θ_{ij} between all K directions, thus connecting response mismatches to a *specific* geometrical notion of *collinearity* between functions. Concretely we would have that

$$C_{ij} := \cos(\pi P_{ij}) = \cos(\theta_{ij}) = \left\langle \boldsymbol{u}^{(i)}, \boldsymbol{u}^{(j)} \right\rangle;\ i, j = 1, ..., K$$

where the brackets $\langle , \rangle$ denote the standard scalar product. This idea of collinearity certainly helps to get some intuitions, and we have seen that it explains qualitative patterns in global change experiment data. But now we want to go further and provide a stringent test to see if this formalization *quantitatively* holds. If the test is conclusive, this a strong sign that the geometric view truly captures the essence of mismatch structure between ecological functions. The idea is to see if the mismatch patterns of two given functions with all others, can predict the mismatches between them both. This goes as follows: choose a pair of functions, say function 1 and 2. If there really is underlying directions associated to all functions, we may write the direction vectors $\boldsymbol{u}^{(1,2)}$ associated to functions 1 and 2 as linear combinations of the $K-2$ vectors $\{\boldsymbol{u}^{(i)}; i > 2\}$ associated to the remaining functions, plus the part not contained by the hyperplane spanned by $\{\boldsymbol{u}^{(i)}; i > 2\}$. Thus, there should exist two sets of $K-2$ values $\{\lambda_i^{(1,2)}\}_{i>2}$, and two vectors $\boldsymbol{v}^{(1,2)}$ (with norm smaller than 1) such that

$$\boldsymbol{u}^{(1)} = \sum_{i>2} \lambda_i^{(1)} \boldsymbol{u}^{(i)} + \boldsymbol{v}^{(1)},\ \text{and}\ \ \boldsymbol{u}^{(2)} = \sum_{i>2} \lambda_i^{(2)} \boldsymbol{u}^{(i)} + \boldsymbol{v}^{(2)}$$

where $\left\langle \boldsymbol{u}^{(i)}, \boldsymbol{v}^{(1,2)} \right\rangle = 0$ for all $i > 2$. Despite the fact that we do not know any of the direction vectors, we can still compute the values $\{\lambda_i^{(1,2)}\}$ by noticing that, for any $i > 2$

$$C_{i1} = \left\langle \boldsymbol{u}^{(i)}, \boldsymbol{u}^{(1)} \right\rangle = \sum_{j>2} \left\langle \boldsymbol{u}^{(i)}, \boldsymbol{u}^{(j)} \right\rangle \lambda_j^{(1)} = \sum_{j>2} C_{ij} \lambda_j^{(1)}$$

and similarly for function 2. Thus if we define the $(K-2)$-dimensional vectors $\boldsymbol{\lambda}^{(1,2)} = (\lambda_i^{(1,2)})_{i>2}$; $\boldsymbol{c}^{(1,2)} = (C_{i1,2})_{i>2}$; and the $(K-2) \times (K-2)$ matrix $R = (C_{ij})_{i,j>2}$ then we get that $\boldsymbol{c}^{(1,2)} = R\boldsymbol{\lambda}^{(1,2)}$ so that

$$\boldsymbol{\lambda}^{(1)} = R^{-1}\boldsymbol{c}^{(1)};\ \text{and}\ \boldsymbol{\lambda}^{(2)} = R^{-1}\boldsymbol{c}^{(2)}.$$

These operations do require the matrix R to be invertible and moreover, to have an inverse R^{-1} whose norm is not to large, as this would make the inversion highly sensitive, and thus unreliable in practice. This requires that the functions are sufficiently different from one another so that their respective

directions do not too closely align. If those requirements are met, we can then compute the cosine of the angle between the directions associated to function 1 and 2 as

$$\begin{aligned} C_{12} = \left\langle \boldsymbol{u}^{(1)}, \boldsymbol{u}^{(2)} \right\rangle &= \textstyle\sum_{i>2} \lambda_i^{(1)} \left\langle \boldsymbol{u}^{(i)}, \boldsymbol{u}^{(j)} \right\rangle \lambda_j^{(2)} + \left\langle \boldsymbol{v}^{(1)}, \boldsymbol{v}^{(2)} \right\rangle \\ &= \textstyle\sum_{i>2} \lambda_i^{(1)} R_{ij} \lambda_j^{(2)} + \epsilon \end{aligned}$$

where $\epsilon = \left\langle \boldsymbol{v}^{(1)}, \boldsymbol{v}^{(2)} \right\rangle$ is the contribution of the components of the focal pair of functions not contained in the hyperplane ($|\epsilon| < 1$).

$$\begin{aligned} C_{12} &= \left\langle \boldsymbol{\lambda}^{(1)}, R\boldsymbol{\lambda}^{(2)} \right\rangle + \epsilon \\ &= \left\langle \boldsymbol{c}^{(1)}, R^{-1}RR^{-1}\boldsymbol{c}^{(2)} \right\rangle + \epsilon \\ &= \left\langle \boldsymbol{c}^{(1)}, R^{-1}\boldsymbol{c}^{(2)} \right\rangle + \epsilon \end{aligned}$$

The test is then to compare C_{12} with

$$\hat{C}_{12} := \left\langle \boldsymbol{c}^{(1)}, R^{-1}\boldsymbol{c}^{(2)} \right\rangle$$

The first thing to note is that $\hat{C}_{12}$ should take values between -1 and 1, for all pairs of functions. If that is the case, it is already a very good sign. Furthermore, if functions overlap sufficiently to make the term $\left\langle \boldsymbol{v}^{(1)}, \boldsymbol{v}^{(2)} \right\rangle$ small, we may expect, when repeating this procedure over all pairs of functions, to find a strong 1:1 correlation between C_{ij} and $\hat{C}_{ij}$. If that is the case, it shows that the geometrical view not only holds, but also explain the modular mismatch pattern of the data matrix P_{ij}.

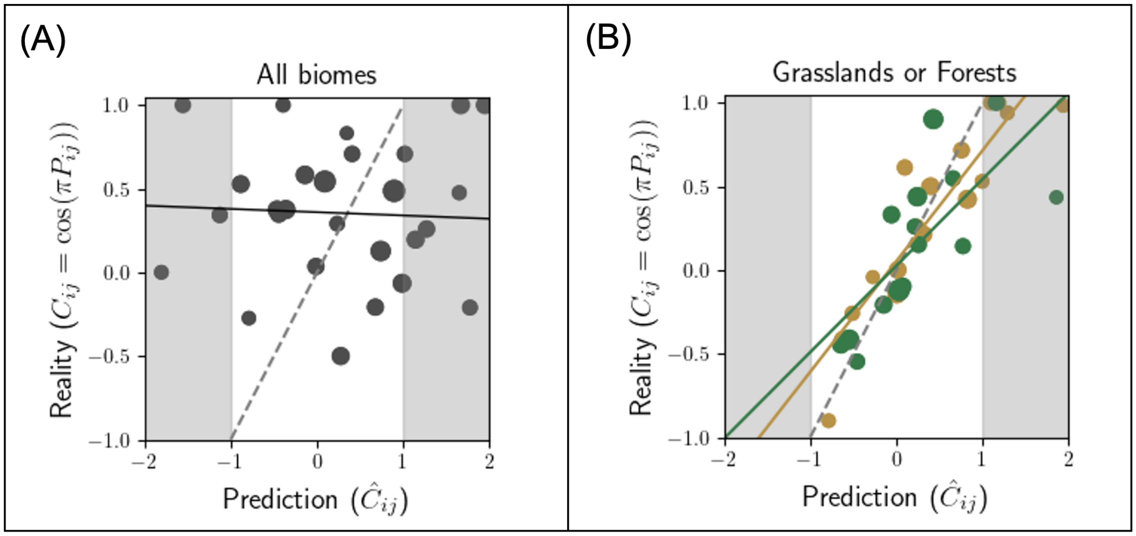

Figure S2: Test of the theory on soil microbial data. **(A)** Data across different biomes are pooled and the test in negative as many points fall out of the acceptable range and no obvious correlation appears. **(B)** After subsetting the data by biome, the test is positive for pairs of functions for which the inverse problem is not too sensitive (here we restrict to $||R^{-1}|| < 20$, but if we lower the bound to 15, all points lie within acceptable bounds). Sizes of points are inversely proportional to the norm of associated R^{-1} matrix. Not all points lie within the acceptable bounds, but most do, in particular those associated with a robust inverse problem.

3 Supplementary Note 3: Collinearity of functions predicted by their estimated broadness

We define the broadness $\mathcal{B}_f$ of an ecosystem function f as the Simpson diversity index $1 \leq {}^2D \leq S$ of the species' per-capita functional contributions, normalized by S. Thus, if the per-capita contributions are denoted ϕ_i, the broadness of the function is

$$\mathcal{B}_f = \frac{{}^2D}{S} = \frac{\left(\sum_i (\frac{|\phi_i|}{\sum_j |\phi_j|})^2\right)^{-1}}{S} = \frac{(\sum |\phi_j|)^2}{S \sum \phi_i^2} = \left(\frac{\langle |\boldsymbol{\phi}|, \mathbf{1}\rangle}{||\boldsymbol{\phi}||\,||\mathbf{1}||}\right)^2$$

So, for a positive (or negative) function ($\boldsymbol{\phi} = \pm|\boldsymbol{\phi}|$) this is the angle made by its direction with the direction $\mathbf{1}$ associated to total biomass. This angle can be related to the mismatch frequency $P_{f,bio}$ between f and total biomass, even if there is a biomass scaling of the perturbation effects (encoded in the diagonal matrix $\Lambda^\alpha = \mathrm{diag}(N_i^\alpha)$). Indeed, without a strong correlation between species realized biomasses and their per-capita functional contributions we get that

$$\cos\Big(\pi P_{f,bio}\Big)^2 = \left(\frac{\Big\langle \boldsymbol{\phi}, \Lambda^{2\alpha}\mathbf{1}\Big\rangle}{||\Lambda^\alpha \boldsymbol{\phi}||\,|\Lambda^\alpha \mathbf{1}||}\right)^2 \approx \left(\frac{\langle \boldsymbol{\phi}, \mathbf{1}\rangle}{||\boldsymbol{\phi}||\,||\mathbf{1}||}\right)^2 = \mathcal{B}_f$$

Thus mismatch patterns with biomass production is a proxy for functional broadness, at least for mostly positive (or negative) functions. We can take this argument a step further to predict the mismatches between functions based on their respective broadness, as would be measured via their response mismatches with biomass production. This reasoning however, relies on the assumption that the functions are uncorrelated to one another, as well as been uncorrelated with the biomass distributions of the systems considered. Thus for two positive functions f and g, associated with directions $\boldsymbol{\phi}$ and $\boldsymbol{\varphi}$, we propose that

$$\cos\Big(\pi P_{f,g}\Big) = \frac{\Big\langle \boldsymbol{\phi}, \Lambda^{2\alpha}\boldsymbol{\varphi}\Big\rangle}{||\Lambda^\alpha \boldsymbol{\phi}||\,||\Lambda^\alpha \boldsymbol{\varphi}||} \approx \frac{\langle \boldsymbol{\phi}, \boldsymbol{\varphi}\rangle}{||\boldsymbol{\phi}||\,||\boldsymbol{\varphi}||} \approx \sqrt{\mathcal{B}_f \mathcal{B}_g}$$

In this view, the mismatch probability between two functions is directly related to the product of their broadness. This statement requires the functions to be positive, but even if they are not, we may still expect that

$$\cos\Big(\pi P_{f,g}\Big) \approx \cos\Big(\pi P_{f,bio}\Big)\cos\Big(\pi P_{g,bio}\Big)$$

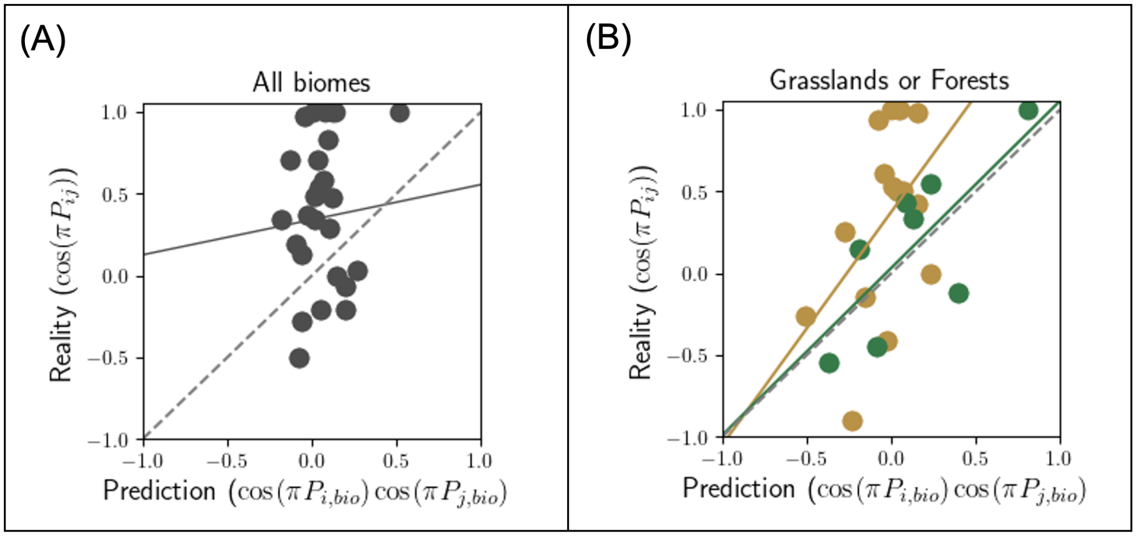

Figure S3: Prediction of mismatches between functions based on their respective mismatches with biomass. **(A)** Data across all biomes are pooled and the prediction fails. **(B)** After grouping the data by biome, although still expected to be a rough approximation, the prediction improves substantially. Points corresponding to cases where one of the function in the pair is biomass have been removed (as they would sit perfectly on the diagonal by construction).
