# Supplementary material for: Variability of functional and biodiversity responses to perturbations is predictable and informative
Source: Nat Commun. 2024 Nov 28;15:10352. doi: 10.1038/s41467-024-54540-3 (PMC11604961; doi:10.1038/s41467-024-54540-3)
Supplement: Supplementary file 2 — Reporting Summary [file 41467_2024_54540_MOESM2_ESM.pdf]

nature portfolio

Corresponding author(s): James Orr

Last updated by author(s): Oct 30, 2024

Reporting Summary

Nature Portfolio wishes to improve the reproducibility of the work that we publish. This form provides structure for consistency and transparency in reporting. For further information on Nature Portfolio policies, see our Editorial Policies and the Editorial Policy Checklist.

Statistics

For all statistical analyses, confirm that the following items are present in the figure legend, table legend, main text, or Methods section.

n/a	Confirmed	
☐	☒	The exact sample size (*n*) for each experimental group/condition, given as a discrete number and unit of measurement
☒	☐	A statement on whether measurements were taken from distinct samples or whether the same sample was measured repeatedly
☒	☐	The statistical test(s) used AND whether they are one- or two-sided<br>*Only common tests should be described solely by name; describe more complex techniques in the Methods section.*
☒	☐	A description of all covariates tested
☐	☒	A description of any assumptions or corrections, such as tests of normality and adjustment for multiple comparisons
☐	☒	A full description of the statistical parameters including central tendency (e.g. means) or other basic estimates (e.g. regression coefficient) AND variation (e.g. standard deviation) or associated estimates of uncertainty (e.g. confidence intervals)
☒	☐	For null hypothesis testing, the test statistic (e.g. *F*, *t*, *r*) with confidence intervals, effect sizes, degrees of freedom and *P* value noted<br>*Give P values as exact values whenever suitable.*
☒	☐	For Bayesian analysis, information on the choice of priors and Markov chain Monte Carlo settings
☒	☐	For hierarchical and complex designs, identification of the appropriate level for tests and full reporting of outcomes
☐	☒	Estimates of effect sizes (e.g. Cohen's *d*, Pearson's *r*), indicating how they were calculated

Our web collection on statistics for biologists contains articles on many of the points above.

Software and code

Policy information about availability of computer code

Data collection	Empirical data were organized in R 4.1.3 using tidyverse 2.0.0, reshape 0.8.9, and plotrix 3.8.
Data analysis	Initial analysis of the empirical dataset was performed using R 4.1.3. Analysis of the simulated data and reanalysis of the empirical data were performed using Python 3.9.7 using numpy, pandas, and matplotlib.<br><br>Our code availability statement is: "The code used in this study is available at https://doi.org/10.5281/zenodo.13985015 . A detailed tutorial with example code is available at https://jamesaorr.github.io/community-properties-tutorial/."

For manuscripts utilizing custom algorithms or software that are central to the research but not yet described in published literature, software must be made available to editors and reviewers. We strongly encourage code deposition in a community repository (e.g. GitHub). See the Nature Portfolio guidelines for submitting code & software for further information.

Data

Policy information about availability of data

All manuscripts must include a data availability statement. This statement should provide the following information, where applicable:

- Accession codes, unique identifiers, or web links for publicly available datasets
- A description of any restrictions on data availability
- For clinical datasets or third party data, please ensure that the statement adheres to our policy

The data used and generated in this study are available at https://doi.org/10.5281/zenodo.13985015 (Orr, Piggott, Jackson, Jackson, & Arnoldi, 2024).

Research involving human participants, their data, or biological material

Policy information about studies with human participants or human data. See also policy information about sex, gender (identity/presentation), and sexual orientation and race, ethnicity and racism.

Reporting on sex and gender	not applicable
Reporting on race, ethnicity, or other socially relevant groupings	not applicable
Population characteristics	not applicable
Recruitment	not applicable
Ethics oversight	not applicable

Note that full information on the approval of the study protocol must also be provided in the manuscript.

Field-specific reporting

Please select the one below that is the best fit for your research. If you are not sure, read the appropriate sections before making your selection.

☐ Life sciences ☐ Behavioural & social sciences ☒ Ecological, evolutionary & environmental sciences

For a reference copy of the document with all sections, see nature.com/documents/nr-reporting-summary-flat.pdf

Ecological, evolutionary & environmental sciences study design

All studies must disclose on these points even when the disclosure is negative.

Study description	In our study we were interested in the variability of community-level responses to a given perturbation. We first analysed a large dataset of global change experiments conducted in microbial soil systems to show that variability of community-level responses is not just random noise, but instead, aggregate properties that are thought to be similar to each other respond to perturbations in the same way. To formalise this intuitive observation, we proposed a theory that quantifies the similarity of aggregate properties and predicts whether they should respond in the same way to a perturbation by taking a geometric approach to the problem. We used simulations to demonstrate that our theory can be used as a null expectation to understand, predict and gain useful information from the variability of community-level responses. We then reanalyzed the empirical data in light of our newly proposed framework.
Research sample	For the synthesis presented in our study, we obtained a dataset of global change experiments conducted in microbial soil systems from Zhou et al., (2020) - https://doi.org/10.1038/s41467-020-16881-7.<br><br>All other data were simulated.
Sampling strategy	We analysed all records in the dataset which included 1235 perturbations from 341 publications.<br><br>We describe the details of the simulations in the section: "Simulation toy model for perturbation experiments" and annotated code is available at: https://doi.org/10.5281/zenodo.13985015.
Data collection	We obtained a dataset of global change experiments conducted in microbial soil systems from Zhou et al., (2020) - https://doi.org/10.1038/s41467-020-16881-7. The data in this dataset came from 341 publications.<br><br>Simulated data were generated using code written in Python 3.9.7 by James Orr and Jean-Francois Arnoldi.
Timing and spatial scale	The dataset we used for our synthesis was published in 2020. A total of 1235 observations of global change factor experiments from 341 publications are available in that dataset. These experiments have a global distribution; there are experiments from every continent (including Antarctica), although there is a strong bias towards North America, Europe, and China. The experiments were

conducted before 2020 with some experiments dating back to the 1990s, however the vast majority of experiments were conducted between 2010 and 2020. Experiments varied in their duration from single seasons to multiple years.

Data exclusions	No data were excluded.
Reproducibility	Annotated code is available so that the study is fully reproducible. The scripts have been succesfully tested on multiple computers and operating systems.
Randomization	Empirical and simulated data were not separated into different groups apart from species.
Blinding	The synthesis was performed using an automated script, so meta-information of each study was not known during the analysis.

Did the study involve field work? ☐ Yes ☒ No

Reporting for specific materials, systems and methods

We require information from authors about some types of materials, experimental systems and methods used in many studies. Here, indicate whether each material, system or method listed is relevant to your study. If you are not sure if a list item applies to your research, read the appropriate section before selecting a response.

Materials & experimental systems

n/a	Involved in the study
☒	☐ Antibodies
☒	☐ Eukaryotic cell lines
☒	☐ Palaeontology and archaeology
☒	☐ Animals and other organisms
☒	☐ Clinical data
☒	☐ Dual use research of concern
☒	☐ Plants

Methods

n/a	Involved in the study
☒	☐ ChIP-seq
☒	☐ Flow cytometry
☒	☐ MRI-based neuroimaging

Plants

Seed stocks	*Report on the source of all seed stocks or other plant material used. If applicable, state the seed stock centre and catalogue number. If plant specimens were collected from the field, describe the collection location, date and sampling procedures.*
Novel plant genotypes	*Describe the methods by which all novel plant genotypes were produced. This includes those generated by transgenic approaches, gene editing, chemical/radiation-based mutagenesis and hybridization. For transgenic lines, describe the transformation method, the number of independent lines analyzed and the generation upon which experiments were performed. For gene-edited lines, describe the editor used, the endogenous sequence targeted for editing, the targeting guide RNA sequence (if applicable) and how the editor was applied.*
Authentication	*Describe any authentication procedures for each seed stock used or novel genotype generated. Describe any experiments used to assess the effect of a mutation and, where applicable, how potential secondary effects (e.g. second site T-DNA insertions, mosiacism, off-target gene editing) were examined.*
